# Cross-Sectional Survey of Acupuncturists in the United States Who Prescribed Chinese Herbal Medicine for Patients with Symptoms Likely Related to COVID-19

**DOI:** 10.1089/jicm.2022.0700

**Published:** 2023-08-09

**Authors:** Belinda J. Anderson, Melissa Zappa, Brent D. Leininger, Lisa Taylor-Swanson

**Affiliations:** ^1^ College of Health Professions, Pace University, New York, New York, USA.; ^2^ Albert Einstein College of Medicine, Bronx, New York, USA.; ^3^ Huntsman Cancer Institute, University of Utah, Salt Lake City, Utah, USA.; ^4^ Integrative Health & Wellbeing Research Program, Earl E. Bakken Center for Spirituality & Healing, University of Minnesota, Minneapolis, Minnesota, USA.; ^5^ College of Nursing, University of Utah, Salt Lake City, Utah, USA.

**Keywords:** COVID-19, Chinese herbal medicine, complementary medicine, pandemic

## Abstract

**Objective:**

The objective of this study was to examine the prescribing of Chinese herbal medicine (CHM) by licensed acupuncturists in the United States during the COVID-19 pandemic.

**Methods:**

A 28-question survey with nine branching questions was disseminated through collegial networks, paid advertisements, and a study website in April–July 2021. Participants indicated that they were licensed acupuncturists who treated more than five patients for symptoms likely related to COVID-19 to gain entry to the full survey. Surveys were undertaken electronically through the Research Electronic Data Capture (REDCap) system.

**Results:**

The survey was undertaken by 103 participants representing all US geographic regions and had an average of 17 years in practice. Sixty-five percent received or intended to receive the COVID-19 vaccine. Phone and videoconference were the predominant methods of patient contact; granules and pill forms of CHM were the most prescribed. A wide variety of information sources were used in devising patient treatments inclusive of anecdotal, observational, and scientific sources. Most patients were not receiving biomedical treatment. Ninety-seven percent of participants reported that they had no patients die of COVID-19, and the majority reported that <25% of their patients developed long hauler syndrome (post-acute sequelae SARS-CoV-2 infection).

**Conclusions:**

: This study demonstrates that licensed acupuncturists were treating COVID-19 infected individuals in the United States during the early stages of the pandemic, and for many such patients this was the only therapeutic intervention they had access to from a licensed health care provider. Information disseminated from China through collegial networks, along with published sources including scientific studies, informed the approach to treatment. This study provides insight into an unusual circumstance in which clinicians needed to establish evidence-based approaches to the treatment of a new disease during a public health emergency.

## Introduction

One of the most concerning aspects of coronavirus disease (COVID-19) during the first year of the pandemic was that there were no biomedical antiviral treatments. In China, use of Traditional Chinese Medicine (TCM) for COVID-19 was undertaken both alone and in combination with biomedical symptomatic treatments,^1,2^ and the Chinese government established a prevention and control strategy based on TCM and biomedicine.^3^

Chinese herbal medicine (CHM) was an important part of this strategy because it had been used extensively for numerous prior epidemics, which also significantly shaped theories and treatment approaches throughout the history of the use of CHM.^2,4–7^ More recently, CHM was successfully utilized during the 2003 SARS epidemic and the 2009–2010 flu season associated with H1N1 virus.^6,8,9^ The broad symptom picture that COVID-19 infected patients can present with are encompassed by TCM diagnostic patterns and organ relationships,^1^ and the use of TCM theories has provided insight into the pathology associated with COVID-19 infection.^10^

East Asian medicine (EAM) has been practiced throughout Asia for thousands of years, and more recently all over the world. EAM encompasses several different modalities, including but not limited to acupuncture, CHM, moxibustion, cupping, and Tui Na (Chinese medical massage). A very large body of clinical research supports the efficacy and effectiveness of acupuncture,^11^ which is now in widespread use throughout the United States and covered by many private insurers and Medicare for lower back pain.^12,13^

A smaller body of research evidence supports the efficacy and effectiveness of the other EAM modalities, including CHM. Development of the knowledge base supporting CHM was largely undertaken in China throughout the last several millennia, and it represents both classical and more modern approaches to the therapeutic use of the various constituents in CHM pharmacopeia.^14^ The modern approach to EAM in China is referred to as TCM and is distinguished from the preceding approaches commonly referred to as classical Chinese medicine.^15^

The CHM pharmacopeia consists of over 500 different constituents, which are largely of plant origin, along with others of animal and mineral origin. The widespread acceptance of CHM was significantly increased following the Nobel Prize award to You-you Tu for the discovery of artemisinin, a therapeutic drug for malaria derived from the plant *Artenisia annua*, which is a constituent of the CHM pharmacopeia. It is estimated that more than one-third of clinical drugs are extracted and/or derived from CHM resources.^16^

The CHM prescriptions usually consist of 3–20 different constituents, which are called herbal formulas.^17,18^ Practitioners often start with a commonly used formula from documented sources (classical or modern) and then modify by adding and/or removing specific constituents such that the final formula is specific for the presenting condition of individual patients.

Choice of formulas and individual constituents is made within the context of East Asian medical diagnostic and treatment theory, along with an understanding of the actions and effects of formulas and constituents. CHM can be taken internally as a decoction (CHM is cooked in water and strained), or a powder (often termed granules) that is dissolved in water, or in pill or tincture form.

Given the use and applicability of TCM for managing the COVID-19 pandemic in China, information about its use, especially the use of CHM, spread rapidly to other countries in the early stages of the pandemic. In the United States, licensed TCM practitioners (usually referred to as licensed acupuncturists) accessed this information through collegial networks, and through information distributed through continuing education providers, CHM companies, and EAM journals.

One significant source of such information was through the Lotus Institute for Integrative Medicine^19^ who also disseminated information from Dr. John Chen, an internationally recognized CHM expert who has authored many textbooks on this topic.^18,20,21^ In the United States, state laws vary as to regulations associated with licensed acupuncturists prescribing CHM. Some states require licensed acupuncturists to pass a certification exam provided by the National Certification Commission for Acupuncture and Oriental Medicine (NCCAOM)^22^ that is specific to CHM. Other states have no regulations associated with prescribing and/or recommending CHM.

By mid-2020, publications started to appear in scientific journals describing observational studies and randomized controlled clinical trials assessing the effectiveness and efficacy of CHM for COVID-19 symptoms. In a systematic review and meta-analysis published in July 2020, Xiong et al.^23^ pooled the data from 18 trials involving 2275 patients and concluded that there was suggestive evidence that CHM may be beneficial for the treatment of COVID-19 through improvements in clinical symptoms, imaging outcomes, laboratory indicators, shortening the course of the disease, and reducing the number of severe cases.

Since that time, many more randomized controlled trials (RCTs) of CHM for COVID-19 symptoms have been published,^2,9^ further suggesting that CHM may be beneficial for treating COVID-19 symptoms.

In many states throughout the United States, licensed acupuncturists were not considered essential workers during the early stages of the COVID-19 pandemic and were required to close their offices due to lockdown mandates. Many continued to provide medical services to their patients through telehealth,^24^ and CHM was one of the main therapeutic interventions that were offered. The importance of their role in providing treatment was accentuated by the lack of effective biomedical treatments and the rapid rise in infections and deaths.

Given that this was a new disease, practice guidelines for using CHM for COVID-19 symptoms were nonexistent at the beginning of the pandemic. This obviously created a significant limitation to an evidence-based approach to treatment. Yet, classical and modern evidence on the use of CHM for infectious disease existed, which informed clinical decision making. Our study was designed to investigate how licensed acupuncturists who were treating patients with symptoms likely related to COVID-19 with CHM made treatment decisions, and how the pandemic impacted their practices.

## Methods

### Survey instrument

We used a cross-sectional study design with the anonymous survey distributed in the Spring/Summer of 2021. The survey questions were devised by the authors through an iterative process of survey draft editing conducted through e-mail and study team meetings. B.J.A. drafted version one of the survey and made two rounds of revisions based on feedback from L.T.-S. Six subsequent rounds of revisions took place in response to feedback from L.T.-S., M.Z., and B.D.L.

Feedback from L.T.-S. and M.Z. was in relation to the EAM content, and from B.D.L. in relation to the appropriateness of question response options for statistical analysis. The final survey instrument (available as a Supplementary Data) was version 9 and consisted of 28 primary questions. Nine of these questions had additional branching questions that depended on the response to the primary question. The survey was pretested by B.J.A., L.T.-S., and M.Z. to ensure that survey construction was accurate in the Research Electronic Data Capture (REDCap) system.

The survey was also pretested by a colleague who is a licensed acupuncturist and an early-stage research investigator. The survey was not psychometrically tested.

Survey entry was restricted to participants who indicated that they were US licensed acupuncturists and had treated more than five patients with symptoms likely related to COVID-19. Initial survey questions collected general demographic information.

The following questions asked about personal infection and vaccine participation, numbers and types of patients treated with symptoms likely related to COVID-19, how patient appointments were conducted, form of CHM prescribed, sources of information used in devising CHM formulas, patients' use of biomedical treatments, duration of CHM treatment, outcome assessment, side effects, difficulty obtaining herbs, and incidence of long-covid and death among patients.

### Participant recruitment

Survey participants were recruited by disseminating survey invitations among the author's colleagues, paid advertisements through Acupuncture Today, and by creating a website (www.teamcovidstudy.com). No compensation was provided to participants.

### Survey implementation

The use and implementation of the survey was approved by the Institutional Review Board of Albert Einstein College of Medicine (Einstein, IRB #2020-12556). Information about the survey and informed consent was presented at the beginning of the survey, and survey participation indicated informed consent. The survey was conducted between April 1st, 2021 and July 20th, 2021. Surveys were undertaken online through the Einstein REDCap system.

### Statistical analysis

We used descriptive statistics to report the number and percentage of responses to individual survey questions. We used Chi-square or Fisher exact tests to assess potential associations between formal research or evidence-based medicine training and (1) influence by scientific studies; (2) use of biomedical journals to inform COVID-19 herb formulations; and (3) use of outcome instruments. Fisher exact tests were used when expected frequencies of individual cells were less than 5.^25^

We used a two-sample *t*-test or analysis of variance (ANOVA) (when more than two groups were present) to assess for differences in years of clinical experience based on self-reported (1) use of clinical experience in COVID-19 herb formulation and (2) modifications to COVID-19 herb formulations. All analyses were conducted using Stata 13.1.^26^

We used the survey guidelines outlined by Gaur et al.^27^ when writing our report.

## Results

The survey was initiated by 125 respondents. Of these, 2 were not licensed acupuncturists in the United States, and 20 had not treated more than 5 patients with symptoms likely related to COVID-19. Of the remaining 103 respondents, 25 partially completed the survey, and 78 completed the survey.

Table 1 presents the demographic data and respondent's personal COVID experience. Fifty-eight percent of the participants had current certification through the NCCAOM, and 8% were previously certified. All four US geographic regions^28^ were represented, with greater representation from the northeast and west. Participants had an average of 17 years in practice (range of 1–40 years). Sixty-eight percent had formal research training completed on average 12 years prior (range 0–38 years).

**Table 1.** Demographic Data and Practitioner's Personal COVID-19 Experience

NCCAOM certification, *n* (%)	
Yes	60 (58.3)
No	35 (34.0)
In past	8 (7.7)
Location of practice, *n* (%)	
Northeast	32 (31.4)
Midwest	14 (13.7)
South	15 (14.7)
West	41 (40.2)
Years in practice, mean (SD) [min–max]	16.9 (10.3) [1–40]
Research training, *n* (%)	
No	25 (32.0)
Yes	53 (68.0)
Years since completion of training, mean (SD) [min–max]	12.1 (9.9) [0–38]
COVID-19 infection of practitioner, *n* (%)	
Yes	17 (16.8)
No	67 (66.3)
Unsure	17 (16.8)
Use of Chinese herbs for infection/possible infection, *n* (%)	
Yes	33 (97.1)
No	1 (2.9)
Received or intend to receive the COVID-19 vaccine, *n* (%)	
Yes	67 (65.1)
No	29 (28.2)
Unsure	7 (6.8)

NCCAOM, National Certification Commission for Acupuncture and Oriental Medicine; SD, standard deviation.

Seventeen percent of the participants had been infected by COVID-19, and 17% had possibly been infected. Of the infected and possibly infected participants, 97% took CHM for their symptoms. Sixty-five percent received or intended to receive the COVID-19 vaccine, and 7% were unsure.

Most of the participants had treated 30 or fewer patients (Table 2). Participants varied considerably as to the proportion of patients who were treated during the acute initial stage of infection, with 13% reporting that all the patients they treated were in this stage, and 15% reporting that <5% of their patients were in this stage.

**Table 2.** Numbers Treated, COVID-19 Testing Status, and Mode of Patient Interaction

Approximate number of patients treated for possible COVID-19 symptoms, *n* (%)	
<10	29 (33.3)
10–20	27 (33.8)
21–30	10 (12.5)
31–40	4 (5.0)
41–50	2 (2.5)
>50	8 (10.0)
Proportion treated during acute initial phase of COVID-19, *n* (%)	
<5%	12 (15.0)
5%–25%	15 (18.8)
26%–50%	11 (13.8)
51%–75%	10 (12.5)
76%–99%	22 (27.5)
All of them	10 (12.5)
Proportion starting treatment without a positive test for COVID-19, *n* (%)	
<5%	33 (41.3)
5%–25%	11 (13.8)
26%–50%	18 (22.5)
51%–75%	7 (8.8)
76%–99%	7 (8.8)
All of them	4 (5.0)
Visit format for patients treated for possible COVID-19 symptoms, *n* (%)	
In-person^a^	21 (17.1)
Phone	45 (36.6)
Videoconference	37 (30.1)
E-mail	18 (14.6)
Combination	30 (24.4)
Impact of COVID-19 on practice for practitioners doing in-person appointments,^b^ *n* (%)	
Never closed	12 (9.8)
Closed and re-opened	24 (19.5)
House calls for COVID symptoms	7 (5.7)
In-person visits for acute COVID symptoms only	1 (0.8)
In-person visits for post-acute COVID symptoms only	21 (17.1)
In-person visits for any COVID symptoms	12 (9.8)
Difficulty obtaining PPE	9 (7.3)

^a^ In-person was in office, curbside, and outside.

^b^ Respondents could choose all that apply.

PPE, personal protective equipment.

Most patients had already tested positive for COVID-19. Interaction with patients was predominantly by phone (37%) or videoconference (30%) (Table 2). Ten percent of the participants never closed their office, 20% closed and later reopened, and 6% made house calls.

Table 3 indicates that the predominant form in which the CHM was taken by patients was granules (44%), followed by pill forms (33%). The duration of treatment was 30 or fewer days, with 11–20 days being the most common. Only two participants reported their patients experiencing side effects associated with the CHM, with the severity being rated as mild. Forty-nine percent of participants had trouble obtaining Chinese herbs.

**Table 3.** Form of Chinese Herbs Prescribed, Duration of Treatment, Incidence of Side Effects, and Difficulty Obtaining Chinese Herbs

Chinese herb formulations prescribed for possible COVID-19 symptoms,^a^ *n* (%)	
Raw herbs	23 (18.7)
Granules	52 (42.3)
Patent formulas (pill)	40 (32.5)
Differed by patient	5 (4.1)
Differed within patient	15 (12.2)
Other^b^	8 (6.5)
Main Chinese herb formulation for COVID-19 symptoms, *n* (%)	
Raw herbs	11 (14.1)
Granules	34 (43.6)
Patent formulas (pill)	26 (33.3)
Other	7 (9.0)
Duration of treatment, *n* (%)	
<10 Days	23 (29.5)
11–20 Days	26 (33.3)
21–30 Days	20 (25.6)
31–60 Days	8 (10.3)
61–120 Days	1 (1.3)
121–180 Days	0 (0)
>6 Months	0 (0)
Side effects from Chinese herbs for COVID-19 symptoms, *n* (%)	
Yes	2 (2.6)
No	76 (97.4)
Proportion with side effects, *n* (%)	
<5%	0
5%–25%	2
26%–50%	0
51%–75%	0
>75%	0
Severity of side effects were mainly,^c^ *n* (%)	
Severe	0
Moderate	0
Mild	2
Difficulty getting Chinese herbs, *n* (%)	
Yes^d^	38 (48.7)
No	40 (51.3)

^a^ Respondents could choose all that apply.

^b^ Other was pre-boiled vacuum packs, tinctures, and cooking recipes.

^c^ Side effects reported were loose stools/constipation, nausea, gagging.

^d^ Increased demand, supply chain and delivery issues.

Table 4 presents data related to the sources of information and their use in devising CHM formulas. Many different sources of information were consulted in addition to their own clinical experience that was used by 50% of the participants. However, only two participants used primarily their own clinical experience alone. High frequency sources were continuing education providers (31%), colleagues (31%), Chinese medicine journals (28%), herbal medicine companies (27%), and notes from continuing education courses (25%).

**Table 4.** Sources of Information Used and Influences in Devising Chinese Herbal Formulas

Sources of information for devising CHM formulas,^a^ *n* (%)	
Text books	38 (30.9)
Notes from coursework	17 (13.8)
Notes from continuing education courses	31 (25.2)
Herbal medicine companies	33 (26.8)
Professional publications	19 (15.5)
Continuing education providers	38 (30.9)
Biomedical journals	19 (15.5)
Chinese medicine journals	34 (27.6)
Colleagues	38 (30.9)
Clinical experience	62 (50.4)
Primarily clinical experience alone	2 (1.6)
Other^b^	9 (7.3)
Use of information from past viral outbreaks to inform CHM prescribing, *n* (%)	
Yes^c^	47 (60.3)
No	31 (39.7)
Use and modification of preexisting CHM prescriptions, *n* (%)	
Mainly formulas that were un-modified from sources created pre-COVID	15 (19.2)
Mainly formulas that were un-modified from sources describing the treatment of COVID infected patients	6 (7.7)
Formulas that were mainly modified from sources created pre-COVID	8 (10.3)
Formulas that were mainly modified from sources describing the treatment of COVID infected patients	14 (18.0)
Mainly formulas that were designed by the provider	10 (12.8)
Combination of above	19 (24.4)
Other^d^	6 (7.7)
Influenced by anecdotal information from China about use of herbs to treat COVID-19?, *n* (%)	
Yes	54 (69.2)
No^e^	24 (30.8)
Influenced by scientific studies examining the use of herbs to treat COVID-19?, *n* (%)	
Yes	47 (60.3)
No	31 (39.7)

^a^ Respondents could choose all that apply.

^b^ Other included research from China, and the use of classical texts.

^c^ 27/47 (56%) used it because there was no specific information about the use of Chinese herbs for COVID at the time.

^d^ Use of patent formulas and classical Chinese herbal medicine formulas.

^e^ Five responded in the open-ended section for this question that they had consulted with mentors, colleagues, or professors who had knowledge of the use of Chinese herbs for COVID-19 in China.

CHM, Chinese herbal medicine.

Sixty percent of the participants used information about the use of CHM for past viral outbreaks (SARS, Zika, Ebola, H1N1). Participants predominantly (24%) used a combination of herbal formulas from different origins. Thirteen percent of providers used formulas they designed themselves. The majority were influenced by the anecdotal information about the use of Chinese herbs to treat COVID-19 patients that came from China (69%), and by the scientific studies examining the use of CHM to treat COVID-19 (60%).

Outcomes associated with CHM treatment were predominantly undertaken through discussions with patients about their symptoms (Table 5). Ninety-seven percent of participants reported that they had no patients die of COVID-19. Most participants reported that 25% or less of their patients developed long hauler syndrome (post-acute sequelae SARS-CoV-2 [PASC] infection). Most of the patients were not also receiving biomedical treatments.

**Table 5.** Treatment Outcomes and Proportion Receiving Biomedical Treatment

How provider assessed herbal medicine treatment outcomes, *n* (%)	
Discussion with patient about symptoms	77 (62.6)
Use of outcome instrument (e.g., MYMOP, pain scale, SF36, etc.)^a^	10 (8.1)
Other^b^	8 (6.5)
Patient deaths due to COVID-19, *n* (%)	
Yes	2 (2.6)
No	76 (97.4)
Percentage of patients with COVID-19 symptoms who developed long hauler syndrome, *n* (%)	
<5%	46 (59.0)
5%–25%	18 (23.1)
26%–50%	6 (7.7)
51%–75%	2 (2.6)
>75%	1 (1.3)
All of them	1 (2.5)
Don't know	4 (5.1)
Patient deaths due to COVID-19, *n* (%)	
Yes	2 (2.6)
No	76 (97.4)
Proportion of patients with COVID-19 symptoms also receiving biomedical treatment, *n* (%)	
<5%	39 (50.0)
5%–25%	18 (23.1)
26%–50%	9 (11.5)
51%–75%	3 (3.8)
>75%	3 (3.8)
All of them	6 (7.7)

^a^ Pain scales—six respondents, pulse oximetry—two respondents, one respondent each reported use of thermometer, tongue and pulse diagnosis, orthopedic testing, SF36, clinical data, chest imaging outcomes, taste testing.

^b^ COVID testing—two respondents, Symptom resolution—three respondents, one respondent each reported tongue and pulse diagnosis, tongue diagnosis, energy levels, facial diagnosis.

There were no significant associations between whether participants had received formal research or evidence-based medicine training and whether they were influenced by the scientific studies, used biomedical journals to inform their creation or choice of CHM formulas, or used outcome instruments to assess the success of the CHM medicine treatments (Table 6). We also found no association between participants' years of clinical experience and whether they used their own clinical experience to devise CHM formulas, or between years of experience and whether they modified CHM formulas or designed their own formulas (Table 7).

**Table 6.** Associations Between Formal Research Training and COVID-19 Practice Behaviors

Practice behaviors	Formal research or evidence-based medicine training?,* n *(%)		*p*-Value (Chi-square or Fisher exact test)
	No	Yes	
Influenced by scientific studies?			
No	13 (42)	18 (58)	
Yes	12 (26)	35 (74)	0.13
Used biomedical journals to inform COVID-19 herb formulations			
No	22 (37)	37 (63)	
Yes	3 (16)	16 (84)	0.10
Used outcome instruments			
No	24 (35)	44 (65)	
Yes	1 (10)	9 (90)	0.16

**Table 7.** Associations Between Years of Clinical Experience and Chinese Herbal Medicine Practice Behaviors

Practice behaviors	Years of clinical experience*^a^*		*p*-Value (*t*-test or ANOVA* F*-test)
	N	Mean (SD)	
Used clinical experience for COVID-19 herb formulations			0.12
No	38	14.8 (10.0)	
Yes	62	18.1 (10.3)	
COVID-19 formulas (Q18)			0.07
Did not modify from other sources	21	13.6 (7.1)	
Modified from other sources	22	19.5 (11.5)	
Designed themselves	10	20.3 (7.5)	

^a^ Number of responses may differ from Table 4 due to missing data on years of clinical experience for some participants.

ANOVA, analysis of variance.

## Discussion

Our survey collected data from 103 licensed acupuncturists with on average 17 years of clinical experience from all geographical regions across the United States. About a third of participants may have been COVID-19 infected themselves, and of these 97% (all but one person) took CHM medicine for their symptoms. Contrary to popular belief,^29^ the majority of participants (65%) had received the COVID-19 vaccine. This exceeded the proportion of the US population that had been vaccinated at the end of the study period—57.2% for a single dose and 50.9% for two doses,^30^ and was similar to the proportion of hospital-based US health care workers that had been vaccinated by that time.^31^

Patients were treated in both the acute initial phase and the later stages of infection, including patients with long hauler syndrome (PASC infection). Most patients were known to have COVID-19 by virtue of a positive polymerase chain reaction or rapid COVID-19 test. Our study suggests that for most of the patients, CHM was the only treatment provided by a licensed health care practitioner whom they had access to.

Seventeen percent of our participants saw patients in person, and 10% never closed their offices. This difference reflects the use of open-air locations, including curbside—treating patients in cars and outdoor clinics, as well as house calls. The most commonly used telehealth mechanism was the phone, which was similar to that reported in a survey of US acupuncturists investigating the use of telehealth during the COVID-19 pandemic.^24^ Their study also reported that of the 1045 respondents, only 103 had prescribed CHM for patients likely infected with COVID-19. This suggests that our sample size, although small, may be representative of the licensed acupuncturists who were prescribing CHM during the pandemic.

Granulated and pill forms of CHM were the most common forms prescribed, as would be expected because these are the easiest and most convenient for patients to consume. As was reported in studies conducted in China,^23^ CHM was usually taken for 20 days or less with little side effects. Almost half of the participants experienced difficulty obtaining the Chinese herbs and this was likely related to greatly increased demand due to usage in China and other Asian countries,^2,9,32^ and pandemic-related interruptions to the supply chain, and delivery services.

Participants reported using a wide variety of different sources of information to inform their thinking around prescribing CHM. Such sources ranged from anecdotal information obtained from colleagues in the United States and other countries, textbooks, continuing education sources, coursework notes, and from both Chinese medicine and biomedical journals. These sources cover all categories in the evidence pyramid^33^ with a predominance for the lower rungs—expert opinions, textbooks, and observational research. Most of the scientific studies that had been published by the end of the study period were case studies.^23,34^ As such, at that time there was limited access to RCTs and systematic reviews.

This situation created a unique set of circumstances regarding practicing evidence-based medicine. When evidence exists, sources highest on the evidence pyramid, RCTs and systematic reviews, are the superior sources. Given that most of the study participants (68%) had had training in research or evidence-based medicine, and that 60% of them were influenced by the scientific studies examining the use of CHM to treat COVID-19, we speculate that had high-quality RCTs and systematic reviews existed they would have been viewed as reliable evidence sources and been utilized.

Given the necessity to use evidence sources lower on the evidence pyramid, our data show a concerted effort by the participants to access as broad a range of information sources as possible. The extensive information about the use of CHM for past viral epidemics including SARS, and H1N1 influenza^6,9^ was used by 60% of the participants. Fifty-six percent of these participants reported that they used this information because there was no specific information about the use of CHM for COVID-19 at the time.

There did not appear to be any association between those that had research and evidence-based medicine training and the use of scientific studies and biomedical journals. This may have been because of the lack of information from scientific sources relative to the large amount of information that was disseminated throughout the United States about the use of CHM to treat COVID-19 infected patients in China.^19^ The latter was also directly applicable to clinical practice, unlike many research studies where internal validity is often more important than external validity.

The lack of clinical applicability to real-world acupuncture practice has been reported as a deterrent to the use of scientific research by licensed acupuncturists in previous studies.^35–37^ The importance of this may have been magnified by the extreme and unusual circumstances associated with the early stages of the pandemic where no biomedical treatments were available.

Half of the participants also used their own clinical experience; an essential component of evidence-based medicine practice, as originally defined by Sackett et al.^38^ However, only two people exclusively used their own clinical experience. The use of clinical experience was also reflected in the majority of the participants either modifying existing CHM formulas or designing their own. This reflects a high level of confidence and experience with designing CHM formulas, as we might expect given that the average time in practice was 17 years.

The death rate of patients treated by the participants appeared very low, with only two participants reporting that patients they had treated died. The development of long hauler syndrome (PASC infection) was also low. The incidence of long-hauler syndrome among COVID-19 infected individuals is estimated to be between 33% and 60%.^39–41^ Our study, along with the systematic reviews and metanalyses published after our study^9,32^ that suggest CHM may be effective for COVID-19, supports the need for further investigation.

### Limitations

There were several limitations to this study. We do not have conclusive data on how many licensed acupuncturists were treating patients with symptoms likely related to COVID-19 with CHM during this stage of the pandemic, and so we are unable to verify how representative our sample was. The survey used in this study was not psychometrically tested.

## Conclusions

Licensed acupuncturists in the United States were prescribing CHM for patients with COVID-19 using information from journal articles, scientific studies, and anecdotal sources, along with their clinical experience. Most acupuncturists in our study received the COVID-19 vaccine. Patient interaction was mainly through telehealth, and prescribed CHM was predominantly taken in granulated form. The acupuncturists in our study demonstrated a keen ability to make use of existing evidence to diligently contribute to a major public health crisis.

## Supplementary Material

Supplemental data

## References

[1] Lee DYW, Li QY, Liu J, et al. Traditional Chinese herbal medicine at the forefront battle against COVID-19: Clinical experience and scientific basis. Phytomedicine 2021;80:153337; doi: 10.1016/j.phymed.2020.153337 33221457 PMC7521884

[2] Lyu M, Fan G, Xiao G, et al. Traditional Chinese medicine in COVID-19. Acta Pharm Sin B 2021;11(11):3337–3363; doi: 10.1016/j.apsb.2021.09.008 34567957 PMC8450055

[3] Sun CY, Sun YL, Li XM. The role of Chinese medicine in COVID-19 pneumonia: A systematic review and meta-analysis. Am J Emerg Med 2020;38(10):2153–2159; doi: 10.1016/j.ajem.2020.06.069 33071103 PMC7342052

[4] Scheid V. Transmitting Chinese medicine: Changing perceptions of body, pathology, and treatment in late imperial China. Asian Med (Leiden) 2013;8(2):299–360. 26869864 10.1163/15734218-12341319 PMC4746752

[5] Liu J, Manheimer E, Shi Y, et al. Chinese herbal medicine for severe acute respiratory syndrome: A systematic review and meta-analysis. J Altern Complement Med 2004;10(6):1041–1051; doi: 10.1089/acm.2004.10.1041 15674000

[6] Luo H, Tang QL, Shang YX, et al. Can Chinese Medicine be used for prevention of corona virus disease 2019 (COVID-19)? A review of historical classics, research evidence and current prevention programs. Chin J Integr Med 2020;26(4):243–250; doi: 10.1007/s11655-020-3192-6 32065348 PMC7088641

[7] Yang Y, Islam MS, Wang J, et al. Traditional Chinese medicine in the treatment of patients infected with 2019-New Coronavirus (SARS-CoV-2): A review and perspective. Int J Biol Sci 2020;16(10):1708–1717; doi: 10.7150/ijbs.45538 32226288 PMC7098036

[8] Zhang B, Liang S, Zhang J, et al. Syndrome in TCM and therapeutic effect of integrated medicine in patients with SARS. Tianjin J Tradit Chin Med 2004;6:462–466.

[9] Zhang T, Li X, Chen Y, et al. Evidence mapping of 23 systematic reviews of Traditional Chinese Medicine combined with Western Medicine Approaches for COVID-19. Front Pharmacol 2021;12:807491; doi: 10.3389/fphar.2021.807491 35197851 PMC8860227

[10] Pang WT, Jin XY, Pang B, et al. [Analysis on pattern of prescriptions and syndromes of traditional Chinese medicine for prevention and treatment of COVID-19]. Zhongguo Zhong Yao Za Zhi 2020;45(6):1242–1247; doi: 10.19540/j.cnki.cjcmm.20200218.502 32281331

[11] Birch S, Lee MS, Alraek T, et al. Overview of Treatment Guidelines and Clinical Practical Guidelines that recommend the use of acupuncture: A bibliometric analysis. J Altern Complement Med 2018;24(8):752–769; doi: 10.1089/acm.2018.0092 29912569

[12] Miller DW, Roseen EJ, Stone JAM, et al. Incorporating acupuncture into American Healthcare: Initiating a discussion on implementation science, the status of the field, and stakeholder considerations. Glob Adv Health Med 2021;10:21649561211042574; doi: 10.1177/21649561211042574 34471570 PMC8404666

[13] Candon M, Nielsen A, Dusek JA. Trends in insurance coverage for acupuncture, 2010–2019. JAMA Netw Open 2022;5(1):e2142509; doi: 10.1001/jamanetworkopen.2021.42509 35019986 PMC8756308

[14] Bensky D, Clavey S, Stöger E. Chinese Herbal Medicine. Eastland Press: Materia Medica; 2004.

[15] Unschuld PU. Medicine in China: A History of Ideas. University of California Press: Oakland, CA; 1985.

[16] Gao R-r, Hu Y-t, Dan Y, et al. Chinese herbal medicine resources: Where we stand. Chi Herbal Med 2020;12(1):3–13; doi: 10.1016/j.chmed.2019.08.004 PMC9476789 36117557

[17] Scheid V, Bensky D, Barolet R. Chinese Herbal Medicine: Formulas & Strategies. Eastland Press: Seattle, WA; 2009.

[18] Chen JK, Chen TT. Chinese Herbal Formulas and Applications: Pharmacological Effects & Clinical Research. Art of Medicine Press: City of Industry, CA; 2009.

[19] eLotus. TCM Resources for Coping with COVID-19; 2021. Available from: https://www.elotus.org/content/tcm-resources-covid-19 [Last accessed: March 3, 2023].

[20] eLotus. John Chen, Pharm.D., Ph.D., O.M.D., L.Ac.; 2022. Available from: https://www.elotus.org/bio/john-chen-pharmd-phd-omd-lac [Last accessed: March 3, 2023].

[21] Chen JK, Chen TT, Crampton L. Chinese Medical Herbology and Pharmacology. Art of Medicine Press: City of Industry, CA; 2004.

[22] National Certification Commission for Acupuncture and Oriental Medicine. National Certification Commission for Acupuncture and Oriental Medicine; 2022. Available from: https://www.nccaom.org/ [Last accessed: March 3, 2023].

[23] Xiong X, Wang P, Su K, et al. Chinese herbal medicine for coronavirus disease 2019: A systematic review and meta-analysis. Pharmacol Res 2020;160:105056; doi: 10.1016/j.phrs.2020.105056 32622723 PMC7331568

[24] Lee TL, Langley BO, Noborikawa J, et al. The Acupuncture and Telehealth Survey: A cross-sectional survey exploring early COVID-19 impacts on the Acupuncture Profession. J Integr Complement Med 2022;28(1):36–44; doi: 10.1089/jicm.2021.0151 35085022 PMC8794008

[25] Bolboacă SD, Jäntschi L, Sestraş AF, et al. Pearson-Fisher Chi-square statistic revisited. Information 2011;2(3):528–545.

[26] Stata Statistical Software: Release 13 [Program]: StataCorp LLC: College Station, TX; 2013.

[27] Gaur PS, Zimba O, Agarwal V, et al. Reporting survey based studies—A primer for authors. J Korean Med Sci 2020;35(45):e398; doi: 10.3346/jkms.2020.35.e398 33230988 PMC7683244

[28] United States Census Bureau. Census Regions and Divisions of the United States: Suitland, MD; 2022. Available from: https://www2.census.gov/geo/pdfs/maps-data/maps/reference/us_regdiv.pdf

[29] Coulter ID, Whitley MD, Khorsan R, et al. Incorporating Complementary and Integrative Health Providers in the Public Health Pandemic Response: Lessons from COVID-19 and Recommendations for the Future from a Multidisciplinary Expert Panel. RAND Corporation: Santa Monica, CA; 2022. 10.7249/rhq-09-04-04 PMC9519115 36238017

[30] Centers for Disease Control and Prevention. COVID Data Tracker: Atlanta, GA; 2022. Available from: https://covid.cdc.gov/covid-data-tracker/#vaccination-trends

[31] Reses HE, Jones ES, Richardson DB, et al. COVID-19 vaccination coverage among hospital-based healthcare personnel reported through the Department of Health and Human Services Unified Hospital Data Surveillance System, United States, January 20, 2021-September 15, 2021. Am J Infect Control 2021;49(12):1554–1557; doi: 10.1016/j.ajic.2021.10.008 34802705 PMC8598683

[32] An X, Zhang Y, Duan L, et al. The direct evidence and mechanism of traditional Chinese medicine treatment of COVID-19. Biomed Pharmacother 2021;137:111267; doi: 10.1016/j.biopha.2021.111267 33508618 PMC7836975

[33] Burns PB, Rohrich RJ, Chung KC. The levels of evidence and their role in evidence-based medicine. Plast Reconstr Surg 2011;128(1):305–310; doi: 10.1097/PRS.0b013e318219c171 21701348 PMC3124652

[34] López-Alcalde J, Yan Y, Witt CM, et al. Current State of Research about Chinese Herbal Medicines (CHM) for the treatment of coronavirus disease 2019 (COVID-19): A scoping review. J Altern Complement Med 2020;26(7):557–570; doi: 10.1089/acm.2020.0189 32589449

[35] Ryan J. The use of evidence in acupuncture clinical practice. Austr J Acupunct Chin Med 2006;1(1):19–23.

[36] Anderson BJ, Jurawanichkul S, Kligler BE, et al. Interdisciplinary relationship models for Complementary and Integrative Health: Perspectives of Chinese Medicine Practitioners in the United States. J Altern Complement Med 2019;25(3):288–295; doi: 10.1089/acm.2018.0268 30523704 PMC6437621

[37] Armour M, Betts D, Roberts K, et al. The role of research in Guiding Treatment for Women's Health: A qualitative study of Traditional Chinese Medicine Acupuncturists. Int J Environ Res Public Health 2021;18(2); doi: 10.3390/ijerph18020834 [published Online First: 20210119] PMC7835913 33478105

[38] Sackett DL, Rosenberg WM, Gray JA, et al. Evidence based medicine: What it is and what it isn't. BMJ 1996;312(7023):71–72; doi: 10.1136/bmj.312.7023.71 8555924 PMC2349778

[39] Taquet M, Dercon Q, Luciano S, et al. Incidence, co-occurrence, and evolution of long-COVID features: A 6-month retrospective cohort study of 273,618 survivors of COVID-19. PLoS Med 2021;18(9):e1003773; doi: 10.1371/journal.pmed.1003773 34582441 PMC8478214

[40] Taquet M, Geddes JR, Husain M, et al. 6-month neurological and psychiatric outcomes in 236 379 survivors of COVID-19: A retrospective cohort study using electronic health records. Lancet Psychiatry 2021;8(5):416–427; doi: 10.1016/s2215-0366(21)00084-5 33836148 PMC8023694

[41] Huang C, Huang L, Wang Y, et al. 6-month consequences of COVID-19 in patients discharged from hospital: A cohort study. Lancet 2021;397(10270):220–232; doi: 10.1016/s0140-6736(20)32656-8 33428867 PMC7833295

