# Supplementary material for: Cross-Sectional Survey of Acupuncturists in the United States Who Prescribed Chinese Herbal Medicine for Patients with Symptoms Likely Related to COVID-19
Source: J Integr Complement Med. 2023 Aug 9;29(8):510–7. doi: 10.1089/jicm.2022.0700 (PMC10457619; doi:10.1089/jicm.2022.0700)
Supplement: Supplemental data [file Supp_Data.docx]

**Cross-sectional survey of acupuncturists in the United States who prescribed Chinese herbal medicine for patients with symptoms likely related to COVID-19**

Anderson et al, submitted to JICM

**Survey Instrument**

1. Are you a licensed acupuncturist is the USA?

(You must answer yes to be eligible for the study. If you select no, the survey will automatically end)

- - Yes
  - No

1. Have you treated more than five patients with symptoms that may be related to COVID-19 with Chinese herbal medicine?

(You must answer yes to be eligible for the study. If you select no, the survey will automatically end)

- - Yes
  - No

1. Are you NCCAOM certified in Chinese herbal medicine? (Choose one)

- Yes
- No
- Previously NCCAOM certified but discontinued active certification

1. Where do you practice as a licensed acupuncturist and herbalist (City and State)?

- City or cities (if more than one): ­­­­­­­________________
- State or states (if more than one): ­­­­­­­________________

1. How long have you been practicing Chinese herbal medicine? (Open ended)

- Years ________ and months _________

1. Did you get infected with COVID-19? (Choose one)

- No
- Yes
  1. Did you take Chinese herbs for the infection?
     - Yes
     - No
- Unsure – had symptoms but did not get tested
  1. Did you take Chinese herbs for the infection?
     - Yes
     - No

1. Have you received the COVID-19 vaccine, or do you intend to receive the vaccine when you are able to get access to it?
   - Yes
   - No
   - Unsure
2. Approximately how many patients have you treated for symptoms that may be related to COVID-19 disease with Chinese herbal medicine? (Choose one)

- Less than 10
- 10-20
- 21-30
- 31-40
- 41-50
- More than 50

1. What proportion of these patients did you treat for what appeared to be the **acute initial infected phase** of COVID-19 (as opposed to lingering post-acute long hauler-type symptoms)?
   - Less than 5%
   - 5-25%
   - 26-50%
   - 51-75%
   - More than 75%
   - All of them
2. Of the patients you treated with symptoms that may be related to COVID-19 disease, approximately what proportion **started** receiving treatments from you **without** having received a positive COVID test? (Choose one)

- Less than 5%
- 5-25%
- 26-50%
- 51-75%
- More than 75%
- All of them

1. Of the patients you treated with COVID-like symptoms who received a COVID-19 test – approximately how many tested positive for COVID-19? (Choose one)

- Less than 5%
- 5-25%
- 26-50%
- 51-75%
- More than 75%
- All of them
- Not sure

1. How did you conduct appointments with patients that you were treating for symptoms that may be related to COVID-19 disease? (Choose **all** that apply)
   - Face-to-face

In seeing patients face-to-face with symptoms that appeared related to COVID-19, please select all that applied to your situation:

(By long hauler we mean – ongoing debilitating COVID-19 symptoms following recovery of the acute phase of the infection)

- - 1. Please select **all** that apply:
       - You did not close your office during the pandemic
       - You closed your office and then reopened later during the pandemic
       - You did house calls for patients with symptoms that may be related to COVID
       - You ONLY treated patients face-to-face that had **acute (potentially infectious)** symptoms that may have been related to COVID
       - You ONLY treated patients face-to-face that had what appeared to be non-infectious post-acute long hauler-type COVID symptoms
       - You treated face-to-face patients in **both** categories - acute and long-hauler symptoms that may be related to COVID
       - Other ways that you interacted face-to-face with patients that had symptoms that may be related to COVID-19, please explain
  - Via phone
  - Via videoconference
  - Via Email
  - A combination of several of the above, but the interaction method was for the most part consistent for each individual patient

In seeing patients face-to-face with symptoms that appeared related to COVID-19, please select all that applied to your situation:

(By long hauler we mean – ongoing debilitating COVID-19 symptoms following recovery of the acute phase of the infection)

- - 1. Please select **all** that apply:
       - You did not close your office during the pandemic
       - You closed your office and then reopened later during the pandemic
       - You did house calls for patients with symptoms that may be related to COVID
       - You ONLY treated patients face-to-face that had **acute (potentially infectious)** symptoms that may have been related to COVID
       - You ONLY treated patients face-to-face that had what appeared to be non-infectious post-acute long hauler-type COVID symptoms
       - You treated face-to-face patients in **both** categories - acute and long-hauler symptoms that may be related to COVID
       - Other ways that you interacted face-to-face with patients that had symptoms that may be related to COVID-19, please explain
  - A combination of several of the above, and the interaction mode was variable even for individual patients
    1. Please explain

In seeing patients face-to-face with symptoms that appeared related to COVID-19, please select all that applied to your situation:

(By long hauler we mean – ongoing debilitating COVID-19 symptoms following recovery of the acute phase of the infection)

- - 1. Please select **all** that apply:
       - You did not close your office during the pandemic
       - You closed your office and then reopened later during the pandemic
       - You did house calls for patients with symptoms that may be related to COVID
       - You ONLY treated patients face-to-face that had **acute (potentially infectious)** symptoms that may have been related to COVID
       - You ONLY treated patients face-to-face that had what appeared to be non-infectious post-acute long hauler-type COVID symptoms
       - You treated face-to-face patients in **both** categories - acute and long-hauler symptoms that may be related to COVID
       - Other ways that you interacted face-to-face with patients that had symptoms that may be related to COVID-19, please explain
  - Other, please explain

1. In what form(s) were the Chinese herbs taken by the patients that you were treating with symptoms that may be related to COVID-19 disease? (Choose **all** that apply)

- Raw herbs
- Granules
- Patent formulas (pills)
- Different forms for different patients, but the form was for the most part consistent for each individual patient
- Different forms for different patients, and the form was sometimes variable for individual patients
  1. Please explain
- Other, please explain

1. What was the **predominant** form of Chinese herbs that you prescribed for your patients with symptoms that may be related to COVID-19 disease? (Choose one)

- Raw herbs
- Granules
- Patent formulas (pills)
- Other, please explain

1. What sources did you use in devising the Chinese herbal formulas for your patients with COVID symptoms? (Choose **all** that apply)

- Herbal medicine text books
- Notes from courses undertaken during a Chinese medicine degree program
- Notes from continuing education courses
- Information specific to COVID-19 from herbal medicine companies
- Information specific to COVID-19 from professional publications like *Acupuncture Today*
- Information specific to COVID-19 from continuing education providers
- Information specific to COVID-19 from biomedical journals
- Information specific to COVID-19 from Chinese medicine journals
- Information specific to COVID-19 from colleagues
- Own clinical experience
- Own clinical experience and NO extensive use of other sources
- Other sources, please explain

1. Did you use information about the use of Chinese herbs that was specific for the treatment of other recent viral outbreaks - SARS, Zika, Ebola, swine flu - in devising the Chinese herbal formulas for your patients with symptoms that may be related to COVID-19 disease?

- No
- Yes
  1. Did you use this information because at the time there was no specific information about the use of Chinese herbs to treat patients with COVID symptoms?
     - Yes
     - No

1. Were the Chinese herbal medicine prescriptions that you prescribed for your patients with symptoms that may be related to COVID-19 disease (Choose one)

- Mainly formulas that you **did not modify** from sources created **pre**-COVID?
- Mainly formulas that you **did not modify** from sources describing the treatment of COVID infected patients?
- Mainly formulas that you **modified** from sources created **pre**-COVID?
- Mainly formulas that you **modified** from sources describing the treatment of COVID infected patients?
- Mainly formulas you designed yourself for individual patients?
- A combination of the above? Please explain
- Other, please explain

1. Did the **anecdotal information** from China about the use of Chinese herbs to treat COVID-19 infected patients that was disseminated in the US influence your treatment strategies of your patients with symptoms that may be related to COVID disease? (Choose one)

By anecdotal we mean based on reports of the benefits of Chinese herbs to treat patients that had not yet been subjected to some type of systematic testing for effectiveness using appropriate research methodology

- Yes
  1. Optional – please share details about your work in relation to this topic?
- No
  1. Optional – please share details about your work in relation to this topic?

1. Did the **scientific studies** – clinical trials and systematic reviews – examining the use of Chinese herbs to treat coronavirus-infected patients that began being published in the scientific literature in 2020 influence your treatment strategies of your patients with symptoms that may be related to COVID-19 disease? (Choose one)

- Yes
  1. Optional – please share details about your work in relation to this topic?
- No
  1. Optional – please share details about your work in relation to this topic?

1. Optional - Please briefly describe how you went about using the above possible various sources – what was your general methodology going from diagnosing to putting together a herbal formula? For example, you may have consulted texts or notes, had discussions with colleagues, used clinical knowledge and experience, looked at scientific studies etc. Please give us a general idea of your approach and method of devising the formula.
2. What proportion of your patients that had symptoms that may be related to COVID disease were **also receiving biomedical treatments** from a licensed biomedical healthcare provider? (Choose one)

- Less than 5%
- 5-25%
- 26-50%
- 51-75%
- More than 75%
- All of them

1. On average how long did your patients with symptoms that may have been related to COVID disease take the Chinese herbs that you prescribed? (Choose one)

- Less than 10 days
- 11-20 days
- 21-30 days (~1 month)
- 31 days - 60 days (~2 months)
- 61 days – 120 days (~4 months)
- 121 days – 180 days (~6 months)
- More than 181 days (>6 months)

1. How did you measure the success of these herbal medicine treatments? (Choose all that apply)

- Through discussion with patients about their symptoms
- Through the use of an outcome instrument (e.g., pain scale, MYMOP, SF36 etc.)
  1. No
  2. Yes
     - With what proportion of your patients did you use an outcome instrument? (Choose one)
       1. Less than 5%
       2. 5-25%
       3. 26-50%
       4. 51-75%
       5. More than 75%
       6. All of them
     - What outcome instruments did you use? (Open-ended)
- Other, please explain

1. Did your patients with symptoms that may be related to COVID-19 disease experience side effects associated with the Chinese herbs you prescribed?

- No
- Yes
  1. What proportion of your patients had side effects? (Choose one)
     - Less than 5%
     - 5-25%
     - 26-50%
     - 51-75%
     - More than 75%
     - All of them
  2. Were the side effects mainly: (Choose one)
     - Severe
     - Moderate
     - Mild
     - Variable in different patients, please explain
  3. What were the side effects that your patients experienced? (choose all that apply)
     - Gas and bloating
     - Stomach ache
     - Loose stools or constipation
     - Nausea
     - Vomiting
     - Gagging
     - Headache
     - Dizziness
     - Excessive thirst
     - Other, please explain

1. Did you have difficulty getting the Chinese herbs that you wanted to prescribe for your patients? (For example, they were sold out, unavailable etc.)
   - No
   - Yes, please explain
2. To your knowledge did any of your patients die of COVID-19? (Choose one)

- No
- Yes
  1. How many? (free text response)

1. What proportion of your patients with symptoms that may be related to COVID disease developed **long hauler** syndrome (ongoing debilitating COVID-19 related symptoms following recovery from the acute phase of the infection?) (Choose one)

- Less than 5%
- 5-25%
- 26-50%
- 51-75%
- More than 75%
- All of them
- Don’t know

1. Have you had formal research and/or evidence-based medicine training as part of a degree program?
   - No
   - Yes
     1. When (year)
     2. Please explain
